# Static intra-access pressure ratio and cardiovascular events in patients undergoing haemodialysis

**DOI:** 10.1038/s41598-020-58190-5

**Published:** 2020-01-23

**Authors:** Hee Jung Jeon, Jieun Oh, Young-Ki Lee, Ajin Cho, Jong Woo Yoon, Hyunsuk Kim, Dong Ho Shin

**Affiliations:** 1 Department of Internal Medicine, College of Medicine, Hallym University, Kangdong Sacred Heart Hospital, 150, Seongan-ro, Gangdong-gu, Seoul, 05355 Korea; 2 Department of Internal Medicine, College of Medicine, Hallym University, Kangnam Sacred Heart Hospital, 1, Singil-ro, Yeongdeungpo-gu, Seoul, 07441 Korea; 3 Department of Internal Medicine, College of Medicine, Hallym University, Chuncheon Sacred Heart Hospital, 77, Sakju-ro, Chuncheon-si, Gangwon-do 24253 Korea

**Keywords:** Cardiology, Haemodialysis

## Abstract

Static intra-access pressure ratio (SIAPR) measurement, using haemodialysis machine transducers, is the vascular access surveillance method in patients undergoing haemodialysis. However, little is known about the relationship between the SIAPR and arterial stiffness, and the clinical usefulness of the SIAPR in predicting cardiovascular events. A total of 209 patients undergoing maintenance haemodialysis were evaluated. The SIAPRs ranged from 0.01 to 0.52 (median: 0.23). When the patients were divided into two groups according to their median of SIAPR, the incidence of previous cardiovascular disease, E/E′ ratio, and brachial-ankle pulse wave velocity were significantly higher in the patients with SIAPRs of ≤0.23 than in those with SIAPRs of >0.23. Conversely, patients with worse comorbid status had a lower SIAPR than patients without it. In the Kaplan-Meier analysis, the cumulative incidence of cardiovascular events was significantly higher in the patients with SIAPRs of ≤0.23 than in those with SIAPRs of >0.23 (P < 0.001). In the multiple Cox regression analysis, an increase in the SIAPR was associated with a reduced risk for cardiovascular events [hazard ratio: 0.36, 95% confidence interval: 0.21–0.60, P = 0.001]. Therefore, a low SIAPR related with arterial stiffness was a predictor for cardiovascular events.

## Introduction

Cardiovascular diseases are the main cause of death in patients undergoing haemodialysis^1^. Increased arterial stiffness, commonly observed in patients receiving haemodialysis, is a known predictor of the induction of these diseases^2–5^. It results from the progression of atherosclerosis with vascular intimal and medial calcifications^6,7^. Pulse wave velocity (PWV), the speed at which the arterial pulse propagates through the circulatory system, is a good indicator of the degree of arterial stiffness^8,9^. Generally, carotid-femoral PWV (cfPWV) measurement is considered to be the current gold standard method for assessing central arterial stiffness^10^. However, because specialised equipment and specially trained staff are needed to measure the cfPWV, the brachial-ankle PWV (baPWV), which predominantly reflects peripheral arterial stiffness, is widely used owing to its simplicity in East Asian countries^11,12^. Its measurement only requires the wrapping of blood pressure cuffs on the four extremities. However, baPWV measurement may not be appropriate for cardiovascular risk assessment because central arterial stiffness is closely associated with cardiovascular events and mortality.

In patients undergoing haemodialysis, maintaining an adequate vascular access is important to reduce morbidity and mortality^13^. Therefore, regular monitoring and surveillance must be conducted to check for vascular access failure early or to detect significant vascular access stenosis early. The National Kidney Foundation’s Kidney Disease Outcomes Quality Initiative (KDOQI) guidelines suggest conducting regular surveillance of vascular accesses^14^. One of the recommended methods of vascular access surveillance is the measurement of the static intra-access pressure ratio (SIAPR), which is the static intra-access pressure normalised to the mean arterial pressure (MAP), using the transducer of haemodialysis machines without additional equipment^14^. Meanwhile, vascular access flow (Qa) can be calculated as follows: Qa = MAP/(R_out_ + R_in_), where R_in_ and R_out_ denote the inflow resistance (i.e. resistance of the flow tract upstream of the venous needle), such as the arterial segment of the access, arterial anastomosis, feeding artery, and arterial tree to the heart, and the outflow resistance (i.e. resistance of the flow tract downstream of the venous needle), such as the venous segment of the access, venous anastomosis, and venous outflow to the heart, respectively^15^. Conversely, the SIAPR can be calculated as follows: Qa × R_out_/MAP and can be expressed as R_out_/(R_out_ + R_in_)^12^. Generally, a high SIAPR is known to be an indirect indicator of vascular access stenosis related to outflow. A low SIAPR in the absence of inflow and outflow stenosis could be attributed to a high resistance to arterial inflow, which is related to increased arterial stiffness. Therefore, prediction of the degree of arterial stiffness using the SIAPR, without additional equipment, might be possible. In this study, we investigated the relationship between the SIAPR and arterial stiffness. In addition, because there is limited knowledge on the clinical usefulness of the SIAPR in predicting cardiovascular events, we evaluated whether the SIAPR had a prognostic value for cardiovascular events compared to known risk factors.

## Results

### Study population

**Figure 1:**
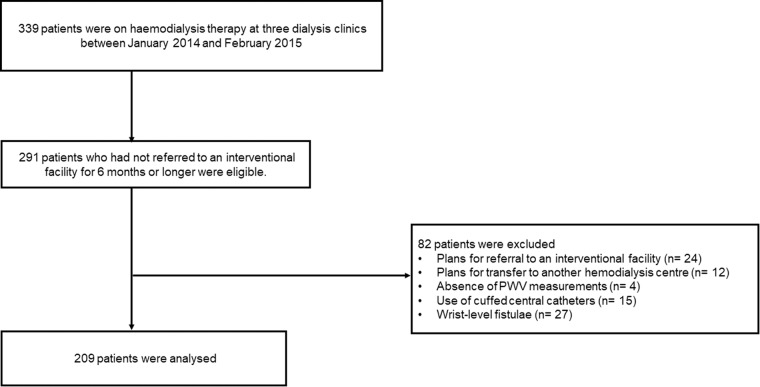
Flow diagram of the study.

A total of 339 patients were on haemodialysis therapy at three dialysis clinics between January 2014 and February 2015. Two hundred ninety-one patients without vascular access dysfunction for 6 months or longer were eligible for inclusion. Eighty-two patients were excluded because of plans for referral to an interventional facility (n = 24), plans for transfer to another haemodialysis centre (n = 12), absence of PWV measurements (n = 4), use of cuffed central catheters (n = 15), and wrist-level fistulae (n = 27). Thus, a total of 209 patients who were on haemodialysis therapy were included in the analysis (Fig. 1).

### Baseline characteristics

**Figure 2:**
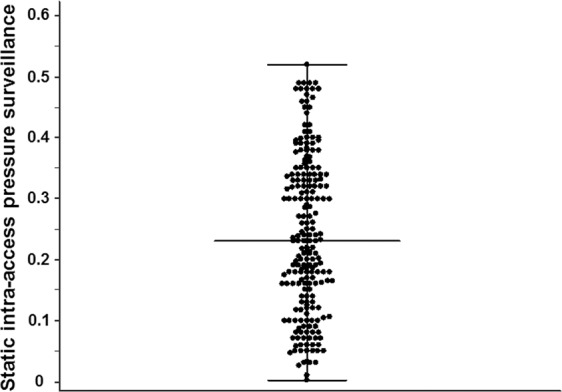
Scattered plots of the SIAPR. Bar and error bar show the median and range, respectively. SIAPR, static intra-access pressure ratio.

Table 1 shows the baseline demographic and clinical data of all patients. The study population consisted of 209 patients undergoing haemodialysis, 99 (47.4%) of whom were men. Their mean age was 59.8 years, and the underlying cause of end-stage renal disease (ESRD) was diabetes in 114 patients (54.5%). The median dialysis duration before study enrolment was 37.4 months. Of note, the SIAPRs ranged from 0.01 to 0.52, with a median of 0.23 (Fig. 2). When the patients were divided into two groups according to their median SIAPR, the incidence of previous coronary artery disease, proportion of left ventricular (LV) diastolic dysfunction, E/E′ ratio, and baPWV were significantly higher in the patients with SIAPRs of ≤0.23 than in those with SIAPRs of >0.23. However, there were no differences between the two groups with respect to age, dry weight, height, MAP, underlying cause of ESRD, vascular access type, medication use, and haemodialysis duration before study enrolment.

**Table 1:** Comparison of the baseline characteristics according to the SIAPR.

Variables	Total (n = 209)	SIAPR of ≤0.23 (n = 104)	SIAPR of >0.23 (n = 105)	P-value
Demographic data				
Age (years)	59.8 ± 11.8	60.6 ± 11.1	58.9 ± 12.4	0.29
Men, n (%)	99 (47.4)	58 (55.8)	41 (39.0)	0.02
Clinical data				
Dry weight (kg)	60.2 (52.0–68.3)	61.3 (53.9–69.4)	59.1 (50.0–67.3)	0.11
Height (cm)	162 (154–168)	163 (154–169)	160 (153–168)	0.26
Systolic blood pressure (mmHg)	150.6 ± 25.0	152.2 ± 21.2	149.0 ± 28.3	0.35
Diastolic blood pressure (mmHg)	77.7 ± 12.1	77.2 ± 11.8	78.2 ± 12.4	0.56
Mean arterial pressure (mmHg)	102.0 ± 14.5	102.2 ± 12.6	101.8 ± 16.2	0.84
Underlying end-stage renal disease cause				
Diabetes, n (%)	114 (54.5)	57 (54.8)	57 (54.3)	0.99
Non-diabetes, n (%)	95 (45.5)	47 (45.2)	48 (45.7)	
Previous cardiovascular disease				
Coronary artery disease	81 (38.8)	56 (53.8)	25 (23.8)	0.001
Peripheral artery disease	12 (5.7)	9 (8.7)	3 (2.9)	0.13
Cerebrovascular disease	48 (23)	29 (27.9)	19 (18.1)	0.13
Vascular access type				
grafts, n (%)	37 (17.7)	13 (12.5)	24 (22.9)	0.08
Arteriovenous fistulae, n (%)	172 (82.2)	91 (87.5)	81 (77.1)	
Medication use				
ß-blockers, n (%)	93 (44.5)	49 (47.1)	44 (41.9)	0.45
RAS blockers, n (%)	130 (62.2)	67 (64.4)	63 (60.0)	0.51
Vitamin D analogues, n (%)	61 (29.2)	34 (32.7)	27 (25.7)	0.34
Calcium-based phosphate binder, n (%)	137 (65.6)	65 (62.5)	72 (68.6)	0.44
Non calcium-based phosphate binder, n (%)	28 (13.4)	13 (12.5)	15 (14.3)	0.86
Antiplatelet drugs use, n (%)	108 (51.7)	56 (53.8)	52 (49.5)	0.63
Duration of haemodialysis^a^ (months)	37.4 (12.6–74.0)	42.0 (11.4–71.5)	30.3 (13.6–74.5)	0.50
Single-pool Kt/V	1.5 (1.4–1.6)	1.5 (1.4–1.6)	1.5 (1.4–1.6)	0.89
baPWV (cm/s)	1935 (1670–2429)	2061 (1825–2595)	1800 (1568– 2300)	<0.001
Echocardiographic parameters				
LVEF (%)	59.3 (53.1–65.0)	56.5 (51.1–63.6)	60.5 (55.5–66.7)	0.01
LVMI (g/m^2^)	135.9 (106.4–164.2)	141.1 (127.3–171.3)	124.1 (97.1–155.6)	0.01
E	77.8 (60.9–103.5)	79.5 (68.5–104.0)	71.1 (58.1–87.8)	0.02
A	82.0 (65.0–95.8)	91.4 (72.7–102.0)	88.5 (74.7–104.0)	0.90
E/A ratio	0.9 (0.7–1.5)	0.8 (0.7–1.1)	0.8 (0.7–0.9)	0.06
DT	201.5 (150.1–248.6)	203.1 (156.5– 251.7)	210.6 (172.0–255.5)	0.28
E/A’ ratio	0.6 (0.5–0.8)	0.6 (0.5–0.7)	0.6 (0.5–0.8)	0.44
E/E’ ratio	18.0 (13.6–24.6)	20.9 (15.4–25.9)	15.4 (12.5–20.5)	<0.001
Left ventricular diastolic dysfunction^b^ (%)	134 (64.1)	81 (77.9)	53 (50.5)	<0.001

Note: Values are expressed as medians ± standard deviations, medians (interquartile ranges), or numbers (percentages).

Abbreviations: SIAPR, static intra-access pressure ratio; RAS, renin-angiotensin system; baPWV, brachial-ankle pulse wave velocity; LVEF, left ventricular ejection fraction; LVMI, left ventricular mass index; A, peak mitral inflow velocities at late diastole; E, peak mitral inflow velocities at early diastole; DT, deceleration time; A′, late diastolic mitral annular velocities obtained on tissue Doppler imaging; E′, early diastolic annular velocities obtained on tissue Doppler imaging.

^a^Before study enrolment.

^b^Left ventricular diastolic dysfunction was defined as an E/E′ ratio of >15.

Mean arterial pressure was calculated by diastolic pressure plus a third of the pulse pressure.

### The SIAPR according to comorbidity

Patients with diabetes [0.22 (0.13–0.32) vs 0.23(0.16–0.37), p = 0.05], previous peripheral artery disease [0.07 (0.04–0.26) vs 0.23 (0.15–0.34), p = 0.01], and previous cerebrovascular disease [0.18 (0.09–0.25) vs 0.29 (0.18–0.35), p = 0.03] had significantly a lower SIAPR than patients without diabetes, previous peripheral artery disease, and previous cerebrovascular disease, respectively.

### Prognostic value of the SIAPR for cardiovascular events

**Figure 3:**
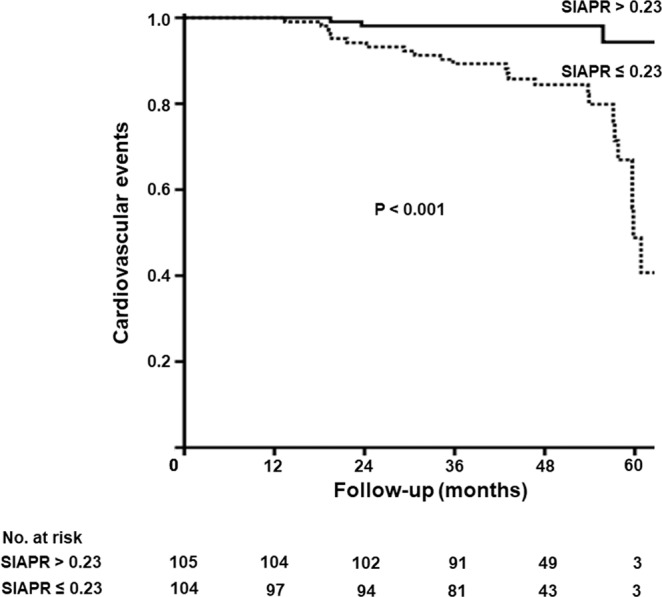
Kaplan-Meier analysis of cardiovascular events according to the SIAPR. The incidence of cardiovascular events was significantly higher in the patients with SIAPRs of ≤0.23 than in those with SIAPRs of >0.23 (P < 0.001). SIAPR, static intra-access pressure ratio.

During a mean follow-up duration of 48.3 months, 27 patients experienced cardiovascular event. Of these, 18, 4, and 5 patients developed coronary artery disease, cerebrovascular disease, and peripheral vascular disease, respectively. Of note, 4 patients died from cardiovascular event. Additionally, of 4 patients with cerebrovascular disease, 1 patient developed another cerebrovascular disease after the event. The incidence of cardiovascular events was significantly higher in the patients with SIAPRs of ≤0.23 than in those with SIAPRs of >0.23 (24 patients, 23.1% vs. 3 patients, 2.9%; P < 0.001). The cumulative probabilities of cardiovascular events were also significantly higher in the patients with SIAPRs of ≤0.23 than in those with SIAPRs of >0.23 (Fig. 3). In the Cox regression analysis, when the SIAPR was considered as a continuous variable, an increase in the SIAPR was associated with a reduced risk for cardiovascular events [hazard ratio (HR): 0.34, 95% confidence interval (CI): 0.21–0.54, P < 0.001]. After adjustment for MAP, previous coronary artery disease, AVG, baPWV, and E/E′ ratio, the SIAPR remained to be associated with cardiovascular events (HR: 0.36, 95% CI: 0.21–0.60, P = 0.001) (Table 2).

**Table 2:** Prediction of cardiovascular events using Cox proportional hazards model.

Variables	Univariate		Multivariate	
	HR (95% CI)	P-value	HR (95% CI)	P-value
Age (per 1 y increase)	1.03 (0.99–1.07)	0.07	1.01 (0.97–1.04)	0.79
Men (vs. women)	1.34 (0.63–2.87)	0.45		
Mean arterial pressure	1.01 (0.98–1.03)	0.82	1.01 (0.98–1.04)	0.48
Diabetes (vs. non-diabetes)	1.09 (0.48–2.45)	0.84		
Previous CAD (vs. previous non-CAD)	3.43 (1.49–7.89)	0.01	1.42 (0.58–3.48)	0.44
AVG (vs. AVF)	2.40 (1.09–5.29)	0.03	3.52 (1.39–8.91)	0.01
ß-blockers use	0.57 (0.26–1.27)	0.17		
RAAS blockers use	0.91 (0.42–1.99)	0.91		
Antiplatelet drugs use	1.52 (0.70–3.33)	0.29		
SIAPR (per 0.1 increase)	0.34 (0.21–0.54)	<0.001	0.36 (0.21–0.60)	0.001
baPWV (per 100 cm/s increase)	1.08 (1.02–1.16)	0.01	0.97 (0.90–1.05)	0.48
E/E’ ratio	1.04 (1.01–1.09)	0.05	1.02 (0.96–1.08)	0.53

Abbreviations: HR, hazard ratio; CI, confidence interval; CAD, coronary artery disease; AVF, arteriovenous fistula; AVG, arteriovenous graft; SIAPR, static intra-access pressure ratio; baPWV, brachial-ankle pulse wave velocity; E, peak mitral inflow velocities at early diastole; E′, early diastolic annular velocities obtained on tissue Doppler imaging.

Mean arterial pressure was calculated by diastolic pressure plus a third of the pulse pressure.

### Additive prognostic value of the SIAPR for the prediction of cardiovascular events

To assess the predictive power of the SIAPR, we calculated Harrell’s C index to be included in the multivariate Cox regression model (Table 3). Compared with the C-statistic of model 1, which included age, MAP, AVG, previous coronary artery disease, baPWV, and E/E′ ratio, the C-statistic (C-statistic: 0.82, 95% CI: 0.70–0.94, P = 0.02) of model 2, in which the SIAPR was added, significantly increased. In addition, model 2 improved the overall continuous net reclassification index (NRI) (NRI: 63.8%, 95% CI 0.04–0.72).

**Table 3:** C-statistics and net reclassification index for the prediction of cardiovascular events.

Model	Harrell C statistic		Continuous NRI
	C-statistics (95% CI)	P-value^a^	Overall (%) with 95% CI
Model 1^b^	0.77 (0.73–0.86)	Ref.	
Model 2^c^	0.82 (0.70–0.94)	0.02	63.8 (0.04–0.72)

^a^P < 0.05: considered significantly different between model 1 and model 2.

^b^Model 1: Age, mean arterial pressure, AVG, previous CAD, baPWV (per 100 cm/s increase), and E/E’ ratio.

^c^Model 2: Model 1 + SIAPR (per 0.1 increase).

Abbreviations: CI, confidence interval; NRI, net reclassification improvement; Ref., reference; AVG, arteriovenous graft; CAD, coronary artery disease; baPWV, brachial-ankle pulse wave velocity; SIAPR, static intra-access pressure ratio.

Mean arterial pressure was calculated by diastolic pressure plus a third of the pulse pressure.

## Discussion

This study is the first to demonstrate that a low SIAPR was significantly associated with cardiovascular events in patients undergoing haemodialysis. Considering that there was the relationship between a low SIAPR and increased arterial stiffness in this study, SIAPR measurement can be a new approach for predicting the development of cardiovascular events in patients undergoing haemodialysis.

Increased arterial stiffness occurs frequently in patients with ESRD^4,5^. In addition, many studies have highlighted the role of arterial stiffness in the development of cardiovascular diseases^3,10,16,17^. Pathophysiologically, increased central arterial stiffness induces afterload of the LV and poor perfusion of the coronary arteries^18^. It results in concentric hypertrophy and fibrosis, which affect LV contraction and relaxation^18^. In fact, previous studies have shown that central arterial stiffness is a strong predictor of cardiovascular mortality in patients with ESRD^17,19^. Of note, there is also enough literature evidence demonstrating that the baPWV is an independent predictor of cardiovascular events and mortality^20–25^. However, theoretically, the baPWV, which reflects peripheral arterial stiffness, is limited in predicting cardiovascular events. In line with these findings, our study showed that although the baPWV was significantly associated with cardiovascular events in the univariate analysis, the HR was too low. In addition, the baPWV was not associated with cardiovascular events in the multivariate analysis.

The vascular access approach for haemodialysis is unique in that it creates a low-resistance connection between the arterial and venous circulations^26^. The haemodynamics of vascular accesses differ according to the access type. While the arterial pressure in an AVG gradually decreases along the length of the graft, the pressure in AVF dissipates within the first few centimetres of the access^14^. Owing to the difference in the pressure profiles of these two access types, the SIAPR in AVF and AVG is known to range from 0.08 to 0.34 and from 0.15 to 0.49, respectively, with reference to the actual pressure at the measurement site divided by the MAP^14^. However, elbow-level fistulae do not have collaterals or have lose collaterals, and often haemodynamically behave like grafts. To reduce the difference in the pressure profile between AVF and AVG, patients with wrist-level fistulae that had collaterals were excluded from the present study. Meanwhile, the SIAPR can be calculated as follows: Q_a_ × R_out_/MAP. In addition, because the Qa can be calculated as MAP/total resistance (R_out_ + R_in_), the SIAPR can be expressed as R_out_/(R_out_ + R_in_). Therefore, a low SIAPR in the absence of inflow and outflow stenosis could be attributed to a high resistance to arterial inflow, which is related to increased arterial stiffness. Thereby, patients with abnormal results of vascular access monitoring or surveillance were excluded from our study. In line with this, we observed that the patients with a low SIAPR had a higher proportion of LV diastolic dysfunction related to arterial stiffness and a higher baPWV than those with a high SIAPR. Conversely, we also showed that patients with worse vascular or comorbid status had a lower SIAPR than patients without it. Of note, although patients with AVG had a higher Charlson Comobidity Index^27^ than patients with AVF (Supplementary Table S1), patients with AVF had a lower SIAPR than patients with AVG due to the difference in the pressure profiles of these two access types [0.21 (0.13–0.33) vs 0.41 (0.14–0.60), p = 0.01]. However, the number of patients with AVG was small in this study. On the other hand, the SIAPR is a direct pressure measurement in AVF or AVG, which depend on the MAP. Thus, after excluding the suspicion for local stenosis, the SIAPR can be influenced directly by cardiac and peripheral blood pressure parameters rather than primarily the elastic properties of the arterial wall. To minimize the effect of overhydration on blood pressure parameters in this study, the measurement of SIAPR was performed after the end of haemodialysis therapy. Interestingly, there was a significant association between the SIAPR and cardiovascular events. Another main finding of this study was that the SIAPR had a predictive value for cardiovascular events compared to known risk factors including MAP.

This study has several limitations. First, this was a small-scale observational study. Second, the SIAPR is less valuable as a tool for assessing arterial stiffness in fistulae. In fistulae, blood entering the venous system returns through multiple collateral veins. However, only elbow- level fistulae without collaterals were included in this study.

In conclusion, because the SIAPR can be measured using the transducer of haemodialysis machines without additional equipment, it may be an attractive approach for predicting cardiovascular events. However, because various clinical conditions need to be considered before this approach can be applied in clinical settings, large-scale observational studies are needed to confirm our findings.

## Methods

### Ethics statement

This study was conducted in accordance with the Declaration of Helsinki principles and approved by the institutional review boards of Kangdong Sacred Heart Hospital, Kangnam Sacred Heart Hospital, and Chuncheon Sacred Heart Hospital (reference nos. 2014-01-025, 2014-04-54, and 2014-96). Written informed consent was obtained from all patients before enrollment.

### Patients

For this prospective observational study, we enrolled patients undergoing haemodialysis at three dialysis clinics (Kangdong Sacred Heart Hospital, Kangnam Sacred Heart Hospital, and Chuncheon Sacred Heart Hospital) between January 2014 and February 2015. Of note, based on these patients’ clinical data, we previously showed the associations between vascular calcification and various clinical characteristics^28,29^. All patients regularly underwent vascular access monitoring and surveillance according to the KDOQI guidelines. Vascular access monitoring (i.e. physical examination to detect vascular access dysfunction) was conducted at least once a week by qualified staff. The SIAPR, a special parameter for detecting vascular access dysfunction, was measured every 4 weeks for vascular surveillance. In addition, the patients were screened for abnormal test results suggesting vascular access dysfunction, such as elevated venous pressure, decreased blood flow, unexplained reduction in Kt/V (K = dialyser urea clearance, t = length of the dialysis session, V = urea distribution volume), or elevated negative arterial prepump pressures^11^.

Patients were eligible if they had undergone haemodialysis without vascular access dysfunction for 6 months or longer. Of note, patients with vascular access dysfunction defined as those who referred to an interventional facility because of abnormal results of vascular access monitoring or surveillance. Patients were excluded if they fulfilled any of the following criteria: 1) plan for referral to an interventional facility because of abnormal results of vascular access monitoring or surveillance, 2) plan for transfer to another haemodialysis centere, 3) absence of PWV measurements, 4) use of cuffed central catheters, and 5) wrist-level fistulae.

### Definitions

Cardiovascular disease was defined as a history of coronary artery disease, cerebrovascular disease, or peripheral vascular disease. Coronary artery disease was defined as myocardial infarction or angina; peripheral artery disease was defined as claudication, ischaemic limb loss, and/or ulceration; and cerebrovascular disease was defined as transient ischaemic attack or stroke. Cardiovascular events were defined as coronary artery disease requiring angioplasty or coronary bypass grafting, peripheral artery disease requiring peripheral revascularisation interventions, or cerebrovascular disease diagnosed using imaging studies.

### PWV assessment

The baPWV was measured using Vascular Profiler 1000 (VP-1000; Colin Co. Ltd., Komaki, Japan). Brachial and post-tibia arterial pressure waveforms were stored for 10 s using extremity cuffs connected to a plethysmographic sensor and an oscillometric pressure sensor wrapped around the arm and ankle. The baPWV was automatically calculated from the distance between two arterial recording sites divided by the transit time^28^. The measurement was performed after the end of haemodialysis session.

### Echocardiographic measurements

Comprehensive echocardiographic measurements were performed using an ultrasound machine (Vivid 7; GE Vingmed Ultrasound AS, Horten, Norway) with a 2.5-MHz probe according to the imaging protocol from the American Society of Echocardiography guidelines^30^. LV ejection fraction was estimated using the modified biplane Simpson’s method in apical two chamber and four-chamber views. LV mass was determined using the method described by Devereux *et al*.^31^, and the LV mass index was calculated by dividing the LV mass by the body surface area. Mitral inflow was assessed using Doppler echocardiography from the apical four-chamber view. The mitral inflow profiles were used to measure the peak mitral inflow velocities at early (E) and late (A) diastole, and their deceleration times. Doppler tissue images of the mitral annulus were also obtained. From the apical four-chamber view, the early (E′) and late (A′) diastolic peak velocities were evaluated. LV diastolic dysfunction was defined as an E/E′ ratio of >15^28,32^. Echocardiographic measurements were performed after the end of haemodialysis session.

### Static intra-access pressure surveillance

After assuring that the zero setting on the pressure transducers of the dialysis delivery system has been calibrated for an accuracy within ±5 mmHg, the venous drip chamber pressure and the MAP in the arm contralateral to the access were obtained from the digital pressure display of the dialysis machine 20–30 s after turning off the dialysis blood pump. The static intra-access venous pressure was calculated as follows: P_IA_ = P_DC_ + 0.76 dH, where P_IA_ (mmHg) is the static intra-access pressure, P_DC_ (mmHg) is the venous drip chamber pressure, and dH (cm) is the difference in the height between the venous drip chamber pressure transducer and the venous needle in the access. Subsequently, the P_IA_ was normalised to the MAP^11^. The measurement was performed at the end of haemodialysis session.

### Data collection, exposure, and outcome determination

The baseline characteristics, including demographic and clinical data, were obtained from medical records at the time of PWV measurement. The exposure for this study was the SIAPR within two weeks before and after PWV measurement. In addition, the outcome was cardiovascular events that occurred after PWV measurement. Of note, all enrolled patients were followed up until the day of cardiovascular events, loss to follow-up, death, or January 2019, whichever occurred first.

### Statistical analyses

All statistical analyses were performed using the Stata software (version 11.0: StataCorp LP, College Station, TX, U.S.A). Continuous variables were expressed as means ± standard deviations or medians (interquartile ranges) and categorical variables as numbers (percentages). The Kolmogorov-Smirnov test was used to analyse the normality of the distribution of parameters among continuous variables. To compare the differences between the groups, Student’s t-test, Mann-Whitney test, the χ² test, or Fisher’s exact test was used. The cumulative incidence of cardiovascular events was calculated using the Kaplan-Meier product estimation method. The independent prognostic values for the study outcome were analysed by performing a Cox regression analysis. A multivariate analysis, which included all of the covariates with P values of <0.1 in the univariate analysis, was performed. Even though a P-value was ≥0.1, potential confounding factor that was known as significant prognostic determinant of cardiovascular events was included in the multivariate analysis. Using Harrell’s C index and the continuous NRI, we determined whether exposure had an additive value compared to the risk factors.

## Supplementary information

Comorbid conditions of the study population according to vascular access type.

## Data Availability

The datasets generated during and/or analysed during the current study are available from the corresponding author on reasonable request.

## References

[1] Cozzolino M (2018). Cardiovascular disease in dialysis patients. Nephrol. Dial. Transpl..

[2] Wheeler DC (1996). Cardiovascular disease in patients with chronic renal failure. Lancet.

[3] Moody WE, Edwards NC, Chue CD, Ferro CJ, Townend JN (2013). Arterial disease in chronic kidney disease. Heart.

[4] Wang MC, Tsai WC, Chen JY, Huang JJ (2005). Stepwise increase in arterial stiffness corresponding with the stages of chronic kidney disease. Am. J. Kidney Dis..

[5] Mourad JJ (2001). Creatinine clearance, pulse wave velocity, carotid compliance and essential hypertension. Kidney Int..

[6] Goodman WG (2004). Vascular calcification in chronic kidney disease. Am. J. Kidney Dis..

[7] Hruska KA, Mathew S, Lund RJ, Memon I, Saab G (2009). The pathogenesis of vascular calcification in the chronic kidney disease mineral bone disorder: the links between bone and the vasculature. Semin. nephrology.

[8] London GM (1990). Aortic and large artery compliance in end-stage renal failure. Kidney Int..

[9] Shoji T (1998). Intermediate-density lipoprotein as an independent risk factor for aortic atherosclerosis in hemodialysis patients. J. Am. Soc. Nephrology: JASN.

[10] Laurent S (2001). Aortic stiffness is an independent predictor of all-cause and cardiovascular mortality in hypertensive patients. Hypertension.

[11] Yamashina A (2002). Validity, reproducibility, and clinical significance of noninvasive brachial-ankle pulse wave velocity measurement. Hypertension research: Off. J. Japanese Soc. Hypertension.

[12] Wang JW, Zhou ZQ, Hu DY (2012). Prevalence of arterial stiffness in North China, and associations with risk factors of cardiovascular disease: a community-based study. BMC cardiovascular Disord..

[13] Santoro A (2000). Confounding factors in the assessment of delivered hemodialysis dose. Kidney Int. Suppl..

[14] Hemodialysis Adequacy Work, G. Clinical practice guidelines for hemodialysis adequacy, update 2006. *Am J Kidney Dis* **48** Suppl 1, S2–90, 10.1053/j.ajkd.2006.03.051 (2006). 10.1053/j.ajkd.2006.03.051 16813990

[15] Spergel LM, Holland JE, Fadem SZ, McAllister CJ, Peacock EJ (2004). Static intra-access pressure ratio does not correlate with access blood flow. Kidney Int..

[16] Cruickshank K (2002). Aortic pulse-wave velocity and its relationship to mortality in diabetes and glucose intolerance: an integrated index of vascular function?. Circulation.

[17] Blacher J (1999). Impact of aortic stiffness on survival in end-stage renal disease. Circulation.

[18] Adenwalla SF, Graham-Brown MPM, Leone FMT, Burton JO, McCann GP (2017). The importance of accurate measurement of aortic stiffness in patients with chronic kidney disease and end-stage renal disease. Clin. kidney J..

[19] Blacher J (2003). Aortic pulse wave velocity index and mortality in end-stage renal disease. Kidney Int..

[20] Sugawara J, Tanaka H (2015). Brachial-Ankle Pulse Wave Velocity: Myths, Misconceptions, and Realities. Pulse.

[21] Ninomiya Toshiharu, Kojima Iwao, Doi Yasufumi, Fukuhara Masayo, Hirakawa Yoichiro, Hata Jun, Kitazono Takanari, Kiyohara Yutaka (2013). Brachial-ankle pulse wave velocity predicts the development of cardiovascular disease in a general Japanese population. Journal of Hypertension.

[22] Takashima N (2014). The relationship of brachial-ankle pulse wave velocity to future cardiovascular disease events in the general Japanese population: the Takashima Study. J. Hum. hypertension.

[23] Munakata M, Konno S, Miura Y, Yoshinaga K (2012). Prognostic significance of the brachial-ankle pulse wave velocity in patients with essential hypertension: final results of the J-TOPP study. Hypertension research: Off. J. Japanese Soc. Hypertension.

[24] Ueki Y (2017). The usefulness of brachial-ankle pulse wave velocity in predicting long-term cardiovascular events in younger patients. Heart Vessel..

[25] Ahn KT (2017). Brachial-ankle PWV for predicting clinical outcomes in patients with acute stroke. Blood Press..

[26] Paulson WD, Jones SA (2004). Hemodynamics of the hemodialysis access: implications for clinical management. Contributions nephrology.

[27] Charlson M, Szatrowski TP, Peterson J, Gold J (1994). Validation of a combined comorbidity index. J. Clin. Epidemiol..

[28] Shin DH (2017). Vascular calcification and cardiac function according to residual renal function in patients on hemodialysis with urination. PLoS one.

[29] Cho A (2017). The relationship between intradialytic hypotension and vascular calcification in hemodialysis patients. PLoS one.

[30] Oh JK (1997). The noninvasive assessment of left ventricular diastolic function with two-dimensional and Doppler echocardiography. J. Am. Soc. Echocardiography: Off. Publ. Am. Soc. Echocardiography.

[31] Devereux RB, Reichek N (1977). Echocardiographic determination of left ventricular mass in man. Anatomic validation of the method. Circulation.

[32] Paulus WJ (2007). How to diagnose diastolic heart failure: a consensus statement on the diagnosis of heart failure with normal left ventricular ejection fraction by the Heart Failure and Echocardiography Associations of the European Society of Cardiology. Eur. heart J..

