# Supplementary material for: Static intra-access pressure ratio and cardiovascular events in patients undergoing haemodialysis
Source: Sci Rep. 2020 Jan 23;10:1084. doi: 10.1038/s41598-020-58190-5 (PMC6978457; doi:10.1038/s41598-020-58190-5)
Supplement: Supplementary file 1 — Comorbid conditions of the study population according to vascular access type. [file 41598_2020_58190_MOESM1_ESM.docx]

**Static intra-access pressure ratio and cardiovascular events in patients undergoing haemodialysis**

Hee Jung Jeon, M.D., Ph.D.; Jieun Oh, M.D., Ph.D.; Young-Ki Lee, M.D., Ph.D.; Ajin Cho, M.D., Ph.D.; Jong Woo Yoon, M.D., Ph.D.; Hyunsuk Kim, M.D.; and Dong Ho Shin, M.D., Ph.D

Table 1. Comorbid conditions of the study population according to vascular access type

| Variables | Total (N=209) | AVF (n=172) | AVG (n=37) | P-value |
| --- | --- | --- | --- | --- |
| Age (years) | 59.8±11.8 | 58.8±11.8 | 64±11.2 | 0.02 |
| Men, n (%) | 99 (47.4) | 90 (52.3) | 9 (24.3) | 0.002 |
| Comorbid disease, n (%) |  |  |  |  |
| MI | 81 (38.8) | 65 (37.8) | 16 (43.2) | 0.54 |
| CHF | 41 (19.6) | 35 (20.3) | 6 (14.6) | 0.57 |
| CVD | 48 (23.0) | 35 (20.3) | 13 (35.1) | 0.05 |
| PAD | 12 (5.7) | 8 (4.7) | 4 (10.8) | 0.14 |
| Dementia | 3 (1.4) | 1 (0.6) | 2 (5.4) | 0.03 |
| Diabetes | 114 (54.5) | 87 (50.6) | 27 (73.0) | 0.02 |
| COPD | 6 (2.9) | 2 (1.2) | 4 (20.8) | 0.001 |
| Ulcer disease | 3 (1.4) | 1 (0.6) | 2 (5.4) | 0.03 |
| Liver disease | 7 (3.3) | 4 (2.3) | 3 (8.1) | 0.08 |
| Age-adjusted CCI | 6 (5–7) | 6 (4–7) | 7 (6–9) | 0.003 |

Note: Values are expressed as medians ± standard deviations or number (percentage).

Abbreviations: AVF, arteriovenous fistula; AVG, arteriovenous graft; MI, myocardial infarction; CHF, congestive heart failure; CVD, cerebrovascular disease; PAD, peripheral artery disease; COPD, chronic obstructive pulmonary disease; CCI, charlson comorbidity index.
